# Topical Corticosteroids and Fungal Keratitis: A Review of the Literature and Case Series

**DOI:** 10.3390/jcm10061178

**Published:** 2021-03-11

**Authors:** Karl Anders Knutsson, Alfonso Iovieno, Stanislav Matuska, Luigi Fontana, Paolo Rama

**Affiliations:** 1 Cornea and Ocular Surface Unit, San Raffaele Scientific Institute, 20132 Milan, Italy; matuska.stanislav@hsr.it (S.M.); rama.paolo@hsr.it (P.R.); 2 Arcispedale Santa Maria Nuova—IRCCS, 42123 Reggio Emilia, Italy; alfonsoiovieno@hotmail.com (A.I.); fontana.luigi@ausl.re.it (L.F.); 3 Department of Ophthalmology, University of British Columbia, Vancouver, BC V6T 1Z, Canada

**Keywords:** fungal keratitis, topical corticosteroids, topical steroids, rebound effect

## Abstract

The management of fungal keratitis is complex since signs and symptoms are subtle and ocular inflammation is minimal in the preliminary stages of infection. Initial misdiagnosis of the condition and consequent management of inflammation with corticosteroids is a frequent occurrence. Topical steroid use is considered to be a principal factor for development of fungal keratitis. In this review, we assess the studies that have reported outcomes of fungal keratitis in patients receiving steroids prior to diagnosis. We also assess the possible rebound effect present when steroids are abruptly discontinued and the clinical characteristics of three patients in this particular clinical scenario. Previous reports and the three clinical descriptions presented suggest that in fungal keratitis, discontinuing topical steroids can induce worsening of clinical signs. In these cases, we recommend to slowly taper steroids and continue or commence appropriate antifungal therapy.

## 1. Introduction

Fungal keratitis is a major cause of blindness, especially in regions with a tropical climate [1]. In South India, one report found that 44% of infectious keratitis cases were caused by fungi [2]; a more recent and larger series in the same region found a prevalence of 36.5% [3,4], and similar epidemiological studies have found different percentages elsewhere: 37.5% in Ghana [5] and 35% in southern Florida [6]. In more temperate areas, the incidence of fungal keratitis is relatively low, ranging from 1% to 5% [7,8]. The incidence and prevalence of fungal keratitis and the spectrum involved vary largely in different countries and even within different regions of the same nation. Variations are related to climate, age, gender, socioeconomic conditions, agricultural activity, and amount of urbanization [4].

Fungal keratitis is complex to diagnose and treat and is often confused for other forms of infectious disease. Diagnosis is frequently delayed due to subtle worsening of the disease and insufficient clinical suspicion in the early stages of infection. Treatment outcomes are inferior to those of bacterial keratitis and there are few commercially available ophthalmic antifungal agents, which are characterized by poor corneal penetration [9,10,11].

The first signs of fungal keratitis are a corneal infiltrate characterized by a dry, raised necrotic surface, feathery margins, and possible satellite lesions. The infiltrate is associated with subtle signs of ocular irritation [4]. Infections of the central cornea tend to be more severe than infections near the limbus [12]. If the infection is left untreated, signs such as hypopyon, neovascularization, worsening of corneal opacity, and corneal perforation may be observed [12].

The current therapy of fungal keratitis is not satisfactory and most antifungal agents are fungistatic, requiring a long time of treatment [1]. The most commonly used drugs are polyenes such as natamycin and amphotericin B and azoles such as fluconazole and voriconazole [1]. Natamycin is considered a primary choice for filamentous fungal infections but is characterized by poor corneal penetration due to its high molecular weight and poor solubility and is mostly effective in cases of superficial keratitis [1,13]. The Mycotic Ulcer Treatment Trial demonstrated superior clinical and microbiological outcomes of natamycin treatment compared to voriconazole treatment in patients affected by fusarium keratitis [14]. Amphotericin B has a wide spectrum of action and covers *Candida* species and *Aspergillus* but is least effective in *Fusarium* [1,11]. Corneal penetration has shown to be poor with an intact epithelium [15]. Fluconazole is considered a topical agent for *Candida* and *Aspergillus*, while a newer drug, voriconazole, shows a broader spectrum of activity against *Candida*, *Aspergillus*, *Scedosporium*, *Fusarium*, and *Paecilomyces* [16,17]. In advanced cases, to overcome the limitation of poor corneal penetration, antifungal drugs may be administered through intracameral or intrastromal injection [18,19,20,21,22]. A recent review reported contrasting results regarding the role of injection of antifungal agents [23]. The authors suggest a role limited to patients on maximal therapy awaiting a surgical procedure. In cases of non-healing fungal keratitis, therapeutic penetrating keratoplasty is the recommended procedure and achieves successful eradication of the infection in 80–90% of eyes [24,25,26].

Local predisposing factors for development of fungal keratitis are trauma (especially with vegetative matter), contact lens wear, and chronic topical steroid use [4]. Other important risk factors associated with the development fungal keratitis include farming activity, diabetes, human immunodeficiency virus status, ocular surface disorders, previous keratoplasty, exposure keratitis, previous ocular surgery, and herpes simplex virus keratitis [4]. Topical steroid use is considered to be a principal factor for development of fungal keratitis. However, percentages are remarkably variable, with authors reporting steroid use as initial therapy in 1–60% of patients affected by fungal keratitis [27,28,29,30,31,32].

## 2. Methods

In this review of the literature, we searched the electronic database PubMed^®^ (National Library of Medicine, Rockville Pike, Bethesda, MD, USA), using search terms related to fungal keratitis and concomitant use of steroids in patients affected by fungal keratitis. The following search terms were utilized: fungal keratitis; corticosteroids fungal keratitis; steroids fungal keratitis. Additional studies were also identified through manual searching of the reference lists of the included studies and reviews.

## 3. Management of Inflammation in Fungal Keratitis: The Role of Steroids

### 3.1. Role of Steroids in The Medical Management of Fungal Keratitis

The use of corticosteroids in the treatment of corneal infections is still debated amongst experts. The causes of structural damage during keratitis are related to direct effects of microorganisms and indirect damage caused by the inflammatory immune reaction. Antimicrobial drugs eliminate infective microorganisms but generally have no other effects on the inflammatory cascade. The rationale of using corticosteroids is to suppress inflammation, and when used in combination with antimicrobial agents, they may theoretically inhibit both causes of damage. However, the inflammatory response to infection is not necessarily detrimental. Even though corneal inflammation may lead to stromal melting, vascularization, and permanent scarring, it may also inhibit replication of infectious agents and limit the extension of infection [33]. When the inflammatory response is attenuated by corticosteroids in combination with an ineffective antimicrobial drug, microorganisms can reach a higher concentration and penetrate to a greater extent throughout the cornea [33]. There is less controversy about the role of corticosteroids in the treatment of fungal keratitis compared to other infections. Historically, three findings support this idea: (1) Frequent identification of corticosteroids as a single risk factor for development of keratitis; (2) Corticosteroids have been utilized to create experimental models of fungal keratitis in animals; (3) Corticosteroids have been found to worsen the course of existing fungal infection [33,34,35,36,37,38,39,40]. Few studies have focused on steroids as a risk factor for worse outcomes in the treatment of fungal keratitis.

Stern et al. reviewed the role of corticosteroids in association with antimicrobial drugs in various forms of infectious keratitis [33]. The authors thoroughly analyzed the objectives and risks of using topical corticosteroids in relation to general principles for the treatment of infectious diseases. They concluded that topical steroids are definitely contraindicated for the treatment of fungal keratitis and present a relative contraindication for *Acanthamoeba* keratitis. Specifically, the authors identified a series of factors that make the use of corticosteroids undesirable. Firstly, fungi replicate more freely when corticosteroids are administered. In a study by O’Day et al., the effect of topical corticosteroids in combination with a variety of antifungal drugs was tested in an experimental model of *Candida albicans* keratitis [41]. With all drugs except amphotericin B, the use of topical steroids either reversed the therapeutic effect of the drug or allowed a greater replication of microorganisms when compared to an untreated control group. Furthermore, immune suppression may be associated with expansion of the infectious process in the deeper layers of the cornea and is particularly undesirable when considering the characteristics of antifungal drugs [33]. As previously mentioned, most antifungals penetrate the cornea poorly and are less effective when the organisms have reached the posterior half of the cornea [33]. The vast majority of antifungals do not reach fungicidal concentrations in the cornea and should be considered fungistatic [33]. According to the authors, suppression of the host immune response in this context is to be considered deleterious, and thus, corticosteroids in the management of fungal keratitis should be contraindicated [33].

In a retrospective study analyzing a cohort of 83 microbiologically proven cases of fungal keratitis in South Korea, previous use of steroids was associated with a deeper corneal infiltrate, worse disease progression, and inferior treatment outcomes [42]. The patients in this study were divided in two groups: patients with prior use of steroids and patients who had no previous use of steroids before diagnosis of fungal keratitis. In the previous steroid group, a higher proportion of patients had deep infiltration (53.3% vs. 32.1%, *p* = 0.057). Findings from this study indicate that infiltrate depth reflects the progression of infection and is, itself, a risk factor for poor penetration of antifungal agents, explaining the worse outcomes in this subgroup. Steroids may be responsible for delay of diagnosis because of their anti-inflammatory properties, are associated with a decreased response of antifungal agents, and are a known independent risk factor for fungal infection. Overall, 28.9% of patients required surgical intervention, with a higher proportion in the prior use of steroids group (43.3% vs. 20.8%, *p* = 0.023). The evisceration/enucleation rate was similar between the two groups. Even though the study was limited by its retrospective design, the clinical significance is important and the authors highlight the risk and side effects of steroid use.

Further evidence comes from a case series published in 1996, demonstrating that association of antibiotic and corticosteroid drops was an additional risk factor for development of *Fusarium* keratitis in traumatized eyes [43]. Despite maximal antifungal therapy, one patient required therapeutic penetrating keratoplasty for impending corneal perforation, one eye progressed to corneal perforation and required evisceration, and the third eye developed a severe form of endophthalmitis, also requiring evisceration. The authors strongly recommended against the unmonitored use of corticosteroids in post-traumatized eyes for the potential risk of developing fungal keratitis.

### 3.2. Role of Steroids after Therapeutic Keratoplasty for Fungal Keratitis

In patients affected by fungal keratitis who do not respond well to topical and systemic antifungal medication, the worsening clinical conditions may lead to corneal perforation, and surgical procedures such as penetrating keratoplasty and lamellar keratoplasty may be required [25,43,44,45,46,47]. Fungal keratitis is responsible for approximately half of all infectious keratitis cases requiring therapeutic penetrating keratoplasty [48]. However, after keratoplasty, immune rejection and recurrence of infection are two complications that often occur [49,50]. Immune rejection after surgical treatment has been reported with rates ranging from 24% to 57%, with most cases being observed within the 6 months post-operative period [50,51,52,53]. Effective control of postoperative inflammation in the context of keratoplasty for corneal fungal infection is complex. Steroids can reduce intraocular inflammation and prevent immune rejection but, conventionally, have not been used in the early post-operative period for the risk of fungal recurrence and aggravation of fungal keratitis [16,49,52,54,55,56,57]. Some studies have reported that steroid administration can be safely initiated 2 weeks after keratoplasty in the absence of clinical signs of recurrence [49,50,51]. A study conducted by Wang et al. demonstrated that steroids may be utilized even one week after keratoplasty [58]. Specifically, the authors adopted a regimen of 0.02% fluorometholone drops 1 week after keratoplasty. If, after two days, there were no signs of recurrence, the steroid was given more frequently—four times per day. The prospective study enrolled 244 eyes. Fungal keratitis recurred in three eyes (1.23%) whereas graft rejection occurred in eight eyes (6.78%) of patients treated by penetrating keratoplasty and did not occur in patients treated by lamellar keratoplasty. The authors conclude that steroid use at 1 week after keratoplasty is safe and can control intraocular inflammation, with a very low rate of fungal keratitis recurrence. Nonetheless, they suggest that in cases where the complete removal of microorganisms is uncertain, topical steroids should only be utilized after the patient has been treated with appropriate antifungal medication for at least 2 weeks, with no trace of recurrent infection.

### 3.3. Discontinuation of Steroids in Patients Affected by Fungal Keratitis

In light of the previously reported evidence, steroid discontinuation is considered mandatory in cases of suspect or culture-proven fungal keratitis. However, there is no clinical consensus regarding how steroids should be discontinued. There is anecdotal evidence indicating that discontinuing topical steroids abruptly in patients affected by fungal keratitis could cause worsening of the clinical scenario [59]. In their work, Peponis et al. reported two cases of worsening after abrupt cessation of topical steroids. In both patients, the corneal infections worsened in two days, leading to perforation, and emergency keratoplasty was required. The authors concluded that corticosteroids should be tapered to allow antifungal agents to act and the host immune mechanisms to take control of the inflammatory responses. Our group has observed similar clinical findings which confirm the aforementioned observations. We hereby report three cases that highlight the potential negative effect of steroid discontinuation in patients affected by fungal corneal infection.

## 4. Case Series: Detrimental Effects of Abrupt Discontinuation of Topical Steroids in Fungal Keratitis

### 4.1. CASE 1

A 67-year-old woman presented with recalcitrant left contact lens-related keratitis that started 4 months before. Corneal scraping had been performed twice before and yielded negative results. The patient had been treated with topical and oral acyclovir without success and was currently on topical anti-amoebic and corticosteroids (polihexamethylene biguanide (PHMB) 0.02%, betamethasone 0.2% first, and then fluorometholone 0.1% on a tapering course).

**Figure 1:**
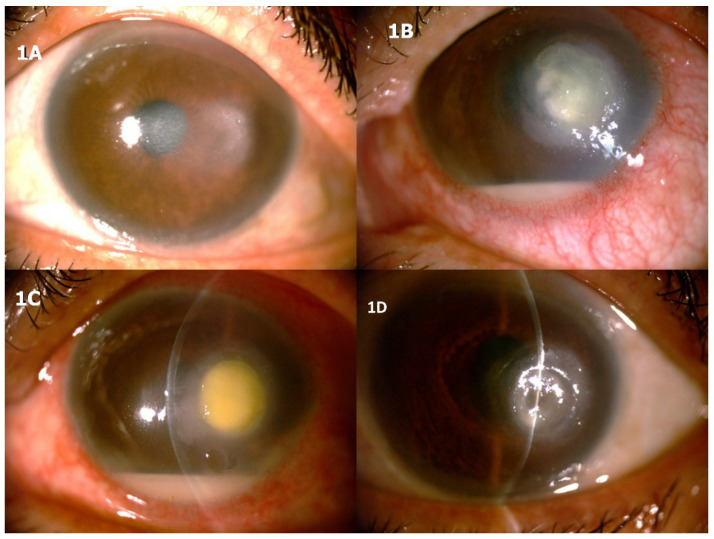
The first patient presented with contact lens-related infectious keratitis. (**A**) After obtaining a positive microscopic examination with identification of hyphae, topical steroids were discontinued immediately and antifungal therapy was started. In the following days, keratitis became more severe (**B**,**C**) and the fungal pathogen was identified as *Beauveria bassiana* and ultimately resolved (**D**) by modifying antifungal therapy.

On slit lamp examination, there was subepithelial haze with a temporal crescent-shaped anterior stromal infiltrate in the absence of epithelial defects (Figure 1A). A third corneal scraping was carried out which, on the following day, showed hyphal elements on Giemsa staining. Consequently, a decision was made to abruptly discontinue topical steroids and start the patient on topical voriconazole 1% hourly and levofloxacin 0.5% four times daily. One week later, the patient reported a significant worsening of symptoms with moderate perikeratic injection, with a deepened enlarged corneal infiltrate with feathery borders and an overlying epithelial defect of approximately the same size, along with an endothelial plaque, anterior chamber 1+ cells and flare, and a 1.5-mm hypopyon (Figure 1B). The fungal pathogen was further identified as *Beauveria bassiana*, which was also found to be resistant to voriconazole. Treatment with topical and intrastromal voriconazole 1%, topical amphotericin B (0.15%) every 2 h, and oral posaconazole 200 mg/day was commenced. During the course of the treatment, the patient also developed an *Escherichia coli* superinfection (Figure 1C, notice round suppurative corneal infiltrate) and ultimately healed in about one and a half months (Figure 1D) after the addition of specific antibiotic therapy.

### 4.2. CASE 2

**Figure 2:**
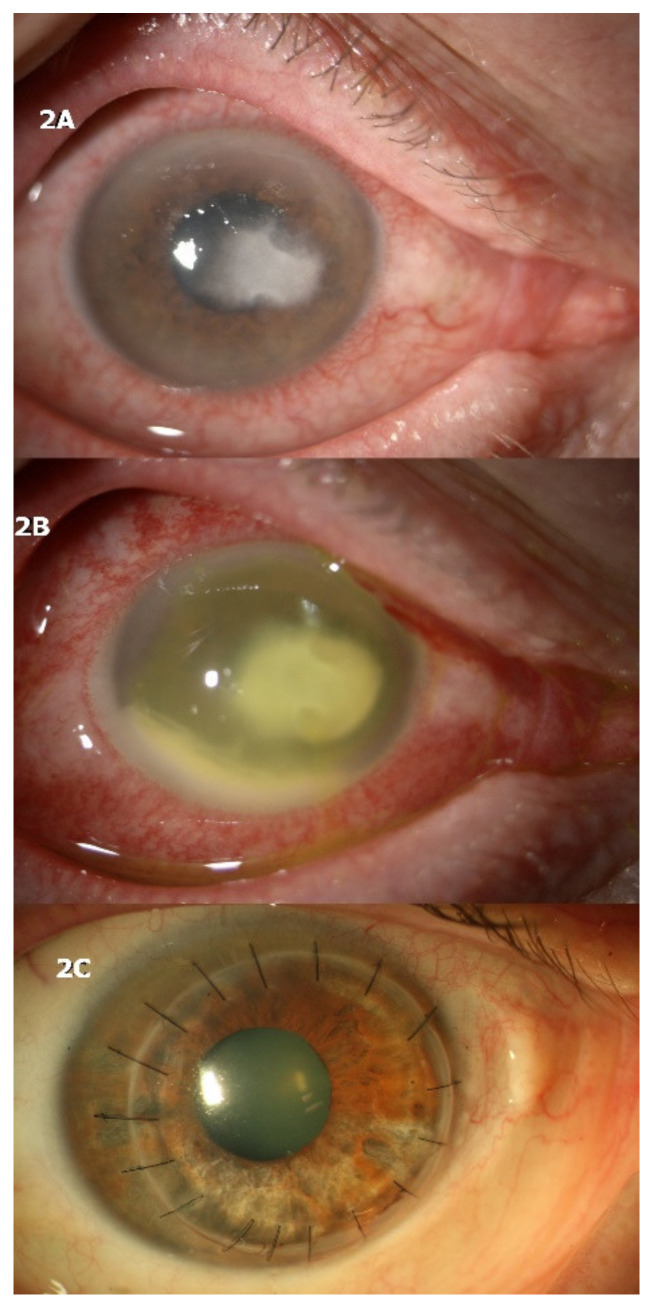
The second patient, also affected by contact lens-related infectious keratitis, was diagnosed with filamentous fungi infection. (**A**) When steroid therapy was discontinued abruptly, severe worsening of clinical signs (**B**) required immediate therapeutic penetrating keratoplasty (**C**).

A 60-year-old male was exposed to mud while wearing soft contact lenses for mild myopia and developed keratitis in the left eye two weeks after the initial contamination. He underwent confocal microscopy in another hospital which resulted positive for *Acanthamoeba* cysts and came to our attention while on topical anti-amoebic and corticosteroid therapy without improvement (PHMB 0.02% every 2 h, hexamidine 0.1% every 2 h, and dexamethasone 0.15% four times daily). The eye presented moderate inflammation and a corneal infiltrate with feathery borders (Figure 2A).

Corneal scraping was performed and direct microscopic examination showed positivity for filamentous fungi. Topical antimycotic and antibiotic therapy with hourly natamycin 5% and moxifloxacin 0.5% four times daily was started, while all other drops were discontinued. On examination 4 days after, there was severe worsening of symptoms and ocular redness, with an increase in the size and depth of the corneal infiltrate and development of hypopyon (Figure 2B). The patient was immediately admitted and therapeutic penetrating keratoplasty was performed 2 days after.

Histology and microbiology examinations resulted positive for *Fusarium* and *Acanthamoeba*. Topical antimycotic and anti-amoebic therapy was continued after keratoplasty, with gradual tapering of the drugs. After 3 months, the patient was without antimicrobic therapy, spectacle-corrected visual acuity was 20/30, and no signs of recurrence were present (Figure 2C).

### 4.3. CASE 3

**Figure 3:**
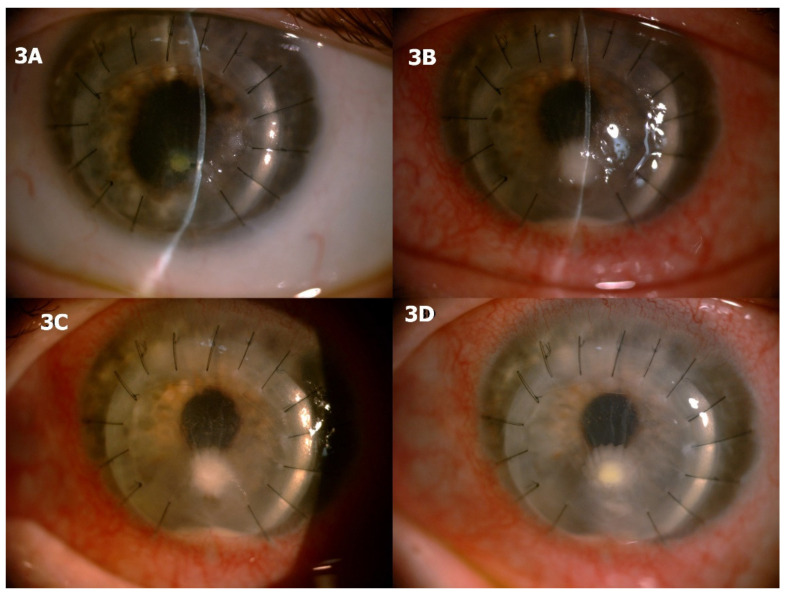
Patient 3 had received previous therapeutic/tectonic keratoplasty for a *Candida albicans* perforated corneal ulcer and presented with fungal keratitis caused by the same pathogen one year later. (**A**) When steroids were discontinued, symptoms and ocular inflammation worsened significantly with hypopyon formation (**B**,**C**). The patient partially improved when appropriate antifungal therapy was commenced and steroids were reintroduced (**D**) but, nevertheless, required a repeat keratoplasty.

A generally healthy 56-year-old woman was brought to our attention for recalcitrant keratitis in her only functional left eye. One year ago, the patient underwent therapeutic/tectonic keratoplasty for a *Candida albicans* perforated corneal ulcer. The patient had been treated elsewhere with bandage contact lens application and topical fluoroquinolones, without success. She presented to our clinic with a paracentral corneal infiltrate with surrounding stromal edema and an overlying same-size epithelial defect at the superior margin of an inferior area of thinning of the corneal graft (Figure 3A). At the time, the patient was on topical moxifloxacin 0.5%, four times daily, and topical loteprednol etabonate 0.5%, three times daily.

The decision was taken to stop the topical corticosteroid and perform a corneal scraping. Topical treatment with fluoroquinolone was continued while awaiting microbiology results. Five days later, the patient sought urgent care for aggravating pain and discomfort. On slit lamp examination, a significant enlargement of the corneal infiltrate and epithelial defect was noted along with marked perikeratic injection, corneal edema, keratic precipitates, and 1.5-mm hypopyon (Figure 3B,C). Microbiology results of the corneal scraping came back positive for *Candida albicans* shortly after. Intensive treatment with hourly amphotericin B 0.15% eye drops was commenced. Topical loteprednol was also sparingly reinstituted (once daily). One week later, the patient reported a net improvement in symptoms. Slit lamp examination displayed a less inflamed eye with significant improvement in corneal infiltrate size, epithelial defect, corneal edema, and hypopyon (Figure 3D). Further improvement was not achieved over the following four weeks of treatment. Therefore, repeat therapeutic keratoplasty was ultimately performed. No recurrent infection was noted over the following three months.

## 5. Discussion (Concluding Remarks)

The management of fungal keratitis is often complex as symptoms are subtle and ocular inflammation is minimal in the initial stages of infection [1]. In our experience at tertiary centers, initial misdiagnosis of the condition and consequent treatment with topical antibiotics and corticosteroids is a frequent occurrence. The most widely used topical antibiotics are fluoroquinolones and aminoglycosides, which have been reported to be effective in some cases of fungal corneal infections [60]. A partial response to empirical antibiotic therapy in fungal ulcers could be one of the reasons prompting the introduction of corticosteroids in these cases. Together with the characteristic slow clinical progression of the infection, it is not uncommon to encounter patients with misdiagnosed fungal ulcers undergoing therapy with topical antibiotics and steroids. Topical steroid use is a well-known risk factor for development of fungal keratitis, with different studies reporting steroid use as initial therapy in 1–60% of patients [27,28,29,30,31,32]. Prolonged use of topical corticosteroids has generally been described to worsen mycotic keratitis and has been attributed to disturbance in the microbial flora of the eye and local immunosuppression [33,61]. Steroids modulate the proliferative response of T-lymphocytes by acting on interleukin 1 and interleukin 2 production and have been used by several investigators to establish animal models of mycotic keratitis [35,36,37,62,63,64]. In a recent study on animal models, the proliferation of *Candida albicans* was higher in a group treated with topical dexamethasone when compared to a control group, and neutrophil infiltration decreased significantly in this group [64]. The authors conclude that corticosteroids exacerbate fungal keratitis not only by determining an increase in fungal aggressivity and a reduction in neutrophil infiltration, but also by inhibiting the formation of neutrophil extracellular traps (NETs). NETs are a first-line defense mechanism aimed at reducing the spread of microorganisms and are formed by the association of chromatin, histones, granular, and cytoplasmic proteins released by neutrophils in the extracellular space [65]. The studies that analyzed the role of steroids as a risk factor for worse outcomes in the treatment of fungal keratitis confirm their detrimental effect and conclude that their discontinuation is considered mandatory in cases of suspect or culture-proven fungal keratitis [33,41,42,43]. However, there are no guidelines regarding how steroids should be discontinued. There is low-grade evidence indicating that discontinuing topical steroids abruptly in patients affected by fungal keratitis could cause worsening of the clinical scenario. These concepts have already been discussed in a smaller case series involving two patients, published by Peponis et al. in 2004 [59]. Both patients required emergency therapeutic keratoplasty for corneal perforation [59]. The small case series reported in the present review adds further evidence and supports the concept that discontinuing topical steroids abruptly can induce worsening of signs and symptoms with an acute rebound inflammatory reaction in patients affected by fungal keratitis. In all of the cases presented, rebound inflammation was observed a few days after steroids were discontinued. One patient required urgent care two days after steroids were stopped and received therapeutic penetrating keratoplasty. The key learning point from these reports is that abrupt steroid discontinuation may lead to the development of unpredictable consequences requiring urgent medical or surgical care. When tapering or discontinuing steroids, patients should be informed of possible worsening and should promptly report changes in signs and symptoms. The physician should be aware that the clinical situation can change rapidly and should be prepared to plan potential surgical procedures. The main limitation of the small series presented in this review is the quality of evidence, which comes from anecdotal case reports. Furthermore, the concepts presented are supported by few similar case reports in the literature [59]. Future investigations should focus on the effect of tapering steroids in patients with fungal keratitis. Ideally, evidence should be gathered from a clinical trial setting, with patients utilizing topical corticosteroids randomized to one of two groups, comparing abrupt discontinuation of steroids vs. slow tapering. Analysis of outcomes should focus on effects such as amount of inflammatory response, successful management of keratitis with medical therapy, and rates of therapeutic keratoplasty or enucleation. According to the limited available evidence in the literature, a common strategy when patients are ultimately diagnosed with fungal keratitis by positive corneal scraping would be to slowly taper steroids, start antifungal therapy, and maintain antibiotic therapy to avoid bacterial superinfection.

## Data Availability

No new data were created or analyzed in this study. Data sharing is not applicable to this article.
